# Risk of Cardiovascular Mortality in Relation to Increased Total Serum IgE Levels in Older Adults: A Population-Based Cohort Study

**DOI:** 10.3390/ijerph16224350

**Published:** 2019-11-07

**Authors:** Kyoung-Bok Min, Jin-Young Min

**Affiliations:** 1 Department of Preventive Medicine, Colleague of Medicine, Seoul National University, Seoul 08826, Korea; minkb@snu.ac.kr; 2 Institute of Health and Environment, School of Public Health, Seoul National University, Seoul 08826, Korea

**Keywords:** immunoglobulin E, premature death, cause of death, cardiovascular disease, population-based cohort

## Abstract

*Background:* Despite a potential link between immunoglobulin E (IgE) levels and cardiovascular disease, the effect of elevated total IgE levels on long-term mortality risk remains unclear. We prospectively investigated the association between total serum IgE levels and all-cause and cardiovascular mortality in US adults. *Methods:* We analyzed data from the 2005–2006 National Health and Nutrition Examination Survey (NHANES) and the NHANES (2005–2006) Linked Mortality Public File. The 2005–2006 NHANES data of 1496 older adults aged ≥50 years and who underwent a serum total IgE antibody test in the initial survey were included. *Results:* After a median follow-up of 119 months, a significant association was observed between total serum IgE levels and cardiovascular mortality, with subjects with the highest total IgE exhibiting a 3.19-fold (HR = 3.19; 95% confidence interval: 1.71–5.96) increase in the risk of cardiovascular mortality compared with those with the lowest total IgE (≤16.80 kU/L). Furthermore, the mortality rate increased with an increase in total IgE levels, regardless of baseline history of cardiovascular diseases (e.g., myocardial infarction, stroke, and noninvasively diagnosed large-vessel peripheral arterial disease). *Conclusions:* This finding suggests that the elevation of IgE levels may be a risk factor for increased cardiovascular mortality.

## 1. Introduction

Immunoglobulin E (IgE) is a class of antibodies synthesized and released by the B lymphocytes of the immune system through interactions among genes, cytokines, and the environment [1]. IgEs can elicit a process of intracellular signaling involving allergens/antigens. Antigens bind to the high-affinity IgE receptor FcεRI on mast cells, basophils, and monocytes [1,2]. They induce degranulation of mast cells and release of a vast array of inflammation mediators [2]. Elevated IgE levels are commonly associated with allergic diseases, such as atopic dermatitis, allergic rhinitis, allergic asthma, and food allergy [3]. Over the past decades, a growing body of evidence has generated new interest in a potential association between total IgE levels and cardiovascular disease [4]. The proposed mechanism is both IgE-independent and -dependent, and involves mast cells that trigger the release of vasoactive inflammation mediators, contributing to the development and progression of myocardial ischemia and thrombosis [5,6,7]. Several cross-sectional studies have demonstrated elevated total serum IgE levels in patients with acute myocardial infarction (AMI), sudden cardiac arrest, and coronary artery disease [8,9,10]. A few prospective studies have suggested elevated total IgE levels as an independent marker of AMI development [11,12]. However, the effect of elevated total IgE levels on long-term mortality risk remains unclear.

Therefore, this study aimed to prospectively investigate the association between total serum IgE levels and all-cause and cardiovascular mortality in the general US population. Furthermore, mortality associated with elevated total IgE levels in the presence and absence of cardiovascular disease at baseline was investigated. 

## 2. Materials and Methods

### 2.1. Study Population

Study data was retrieved from the 2005–2006 National Health and Nutrition Examination Survey (NHANES) and from the NHANES (2005–2006) Linked Mortality Public File from the United States [13]. The NHANES is a series of cross-sectional surveys that employs a stratified, multistage sampling design to obtain data regarding the civilian, non-institutionalized US population. Data from the Linked Mortality File was matched to data of eligible participants retrieved from the NHANES (2005–2006) database using a probabilistic matching algorithm based on the National Death Index until 31 December 2006. The participants’ date and cause of death were obtained from death certificates. Details regarding the survey design and implementation can be obtained on the NHANES 2005–2006 webpage (http://www.cdc.gov/nchs/nhanes.htm).

In total, 2011 participants aged ≥50 years and who underwent a serum total IgE antibody test in the initial survey were included from the 2005–2006 NHANES data and the Linked Morality File. Of these, 515 older adults were excluded because of missing questionnaire information (i.e., health behavior and disease history). The study’s final cohort included 1496 older adults from the 2005–2006 NHANES database.

### 2.2. Measurement of Total Serum IgE Allergen

Serum samples were collected and were analyzed for total IgE allergen using the Pharmacia Diagnostics Immuno CAP 1000 System (Pharmacia Diagnostics, Kalamazoo, MI, USA). A detailed description of laboratory and quality control procedures can be obtained on the NHANES 2005–2006 webpage (http://www.cdc.gov/nchs/data/nhanes/nhanes_05_06/al_ige_d.pdf).

In brief, anti-IgE that was covalently coupled to the ImmunoCap reaction vessel reacted with total IgE in the collected serum sample. Enzyme-labeled anti-IgE antibodies were added to form a complex. Unbound enzyme anti-IgE was washed away, and the bound complex was incubated with developing agent. When the reaction was terminated, fluorescence of the eluate was measured. The fluorescence intensity was proportional to the IgE concentration in the particular sample. The lower limit of detection was 2.00 kU/L.

### 2.3. Identification of Death Cases

International Classification of Diseases 10th Revision (ICD-10) codes were used for all causes of mortality. Based on the underlying causes of mortality from ICD-10, codes I00–I09, I11, I13, I20–I51 were attributed to cardiovascular diseases.

### 2.4. Variables of Interest

Baseline characteristics were retrieved from 2005–2006 NHANES interview data and included age (50–59, 60–69, 70–79, and ≥80 years), sex, ethnic background (White, Black, Hispanic or other), education (below high school, high school graduate, college and higher), family income (<$20,000 or ≥$20,000), smoking status (current, former, or never), and moderate physical activity (yes or no). Body mass index (BMI) was calculated by dividing the estimated weight in kg by the measured height in m^2^ and was categorized in four groups: <18.5 kg/m^2^ (underweight), 18.5–24.9 kg/m^2^ (normal weight), 25–29.9 kg/m^2^ (overweight), and ≥30.0 kg/m^2^ (obese). Regarding disease history, hypertension was defined as systolic blood pressure ≥ 140 mm Hg and diastolic blood pressure ≥ 90 mm Hg, use of antihypertensive drugs or previous physician-diagnosed hypertension. Hypercholesterolemia was defined as a total serum cholesterol level of ≥240 mg/dL or physician-diagnosed hypercholesterolemia. Diabetes was defined as a fasting plasma glucose level of ≥6.99 mmol/L, non-fasting plasma glucose level of ≥11.1 mmol/L, current insulin use, or prior physician diagnosed diabetes. Presence of allergic disease was based on previous allergies, as diagnosed by a physician or health professional.

### 2.5. Statistical Analysis

Significant differences in participants’ characteristics were evaluated using the chi-square test. Total serum IgE levels were categorized into quartiles: Quartile 1 (≤16.80 kU/L), Quartile 2 (16.81–44.10 kU/L), Quartile 3 (44.11–123.00 kU/L), and Quartile 4 (≥123.01 kU/L). After the stratification of total IgE quartiles, Kaplan–Meier curves for cumulative mortality rates were presented according to the total IgE quartiles. The *p*-value was calculated using the log-rank test. The null hypothesis was that there is no difference in survival among IgE quartiles. The log-rank test compares the observed number of deaths in each quartile group and the number expected if the null hypothesis were true. Cox proportional hazard regression was conducted to evaluate the association among total IgE levels and all-cause and cardiovascular mortality. The association between total IgE levels and mortality was further assessed in terms of the presence or absence of baseline cardiovascular diseases, including heart failure, coronary heart disease, heart attack, and angina [14]. Hazard ratios (HRs) and 95% confidence intervals (CIs) were estimated for the outcome variables associated with total IgE quartiles compared with the first quartile. The model’s proportional hazard assumption was confirmed. The regression model was adjusted for all potential confounders including age, sex, race/ethnicity, income, education, smoking status, moderate physical activity, BMI, and history of hypertension, dyslipidemia, diabetes, and allergy.

Weighted estimates of population parameters were computed according to the National Center for Health Statistics to account for the complex sampling design. All analyses were performed using the PROC SURVEY procedures in SAS 9.3 (SAS Institute, Cary, NC, USA) and R (R Foundation for Statistical Computing, Vienna, Austria). Statistical significance was set at α = 0.05 (two-sided).

## 3. Results

Table 1 depicts study participants’ baseline characteristics based on the censored and deceased population. Among 1496 study participants, 431 (28.8%) had died. Compared with the censored subjects, deceased subjects were more likely to be older (the highest proportion in ≥80 years of age), be male, have an income of <$20,000, be educated below high school, be former smokers, be underweight (BMI < 18.5kg/m^2^), and not practice moderate physical activity. Regarding baseline conditions, significant differences were observed between the two groups with respect to the presence of hypertension and diabetes mellitus.

**Figure 1:**
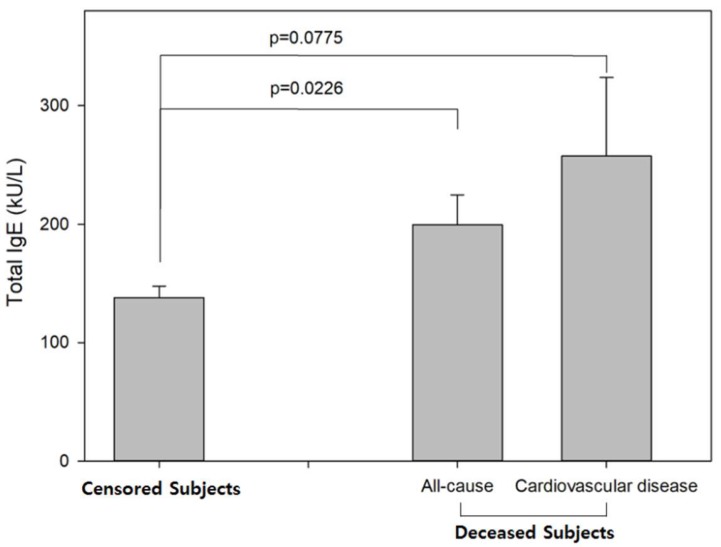
Total serum IgE levels by all-cause and cardiovascular mortality-axis represents participants who were censored (left) and who died (right) because of all-cause and cardiovascular disease. The Y-axis represents total serum IgE levels (kU/L). Bars represent mean value, and error bars represent the standard error. The *p*-value was calculated using the *t*-test.

Figure 1 compares mean total serum IgE levels between censored and deceased. Compared with the mean total IgE levels of censored subjects (149.59 kU/L), those of the deceased subjects were higher (199.56 kU/L; *p* = 0.0226) and were marginally elevated in subjects who die owing to cardiovascular disease (257.46 kU/L; *p* = 0.0775).

**Figure 2:**
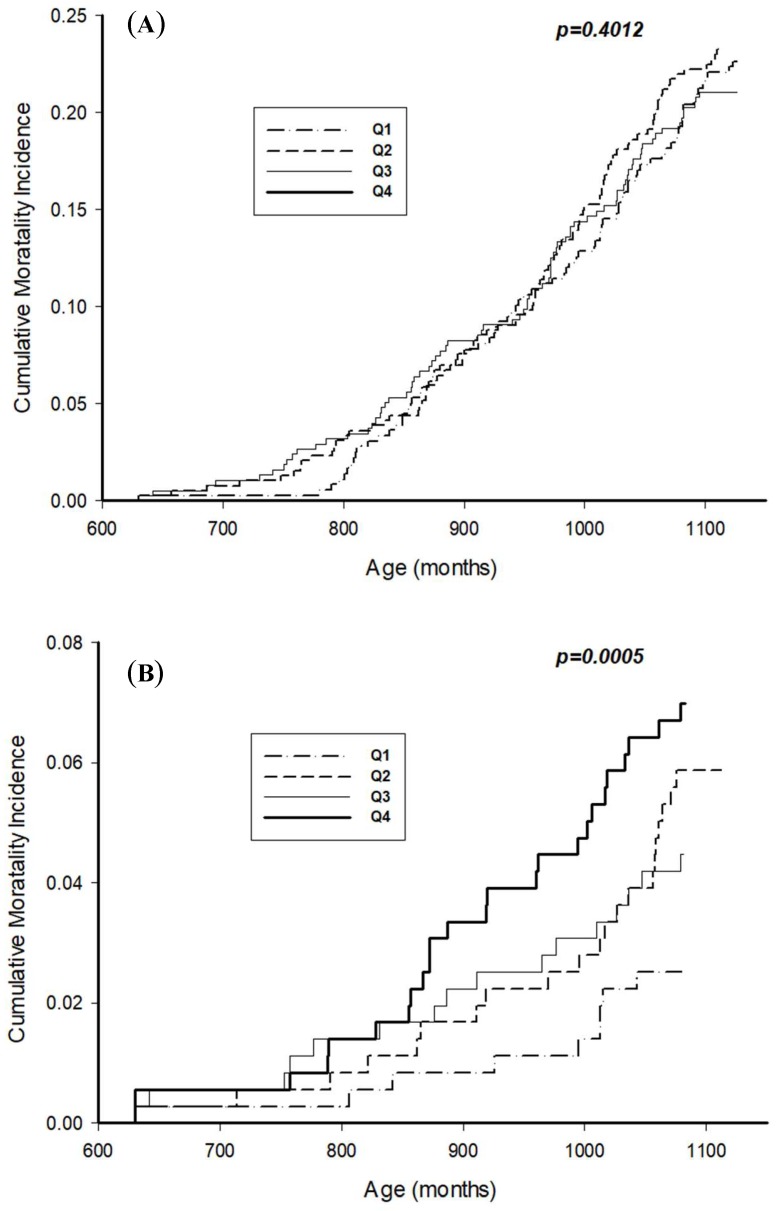
Kaplan–Meier curves for cumulative mortality rates by total IgE quartiles: (**A**) All-cause mortality, and (**B**) cardiovascular mortality. Quartiles of total serum IgE: Q1 (≤16.80 kU/L), Q2 (16.81–44.10 kU/L), Q3 (44.11–123.00 kU/L), and Q4 (≥123.01 kU/L).

Table 2 shows the number of events and adjusted HRs (95% CI) for all-cause and cardiovascular mortality with regard to total IgE quartiles. With increases in the total IgE quartiles, all-cause mortality showed a similar proportion in Quartile 1 (n = 105; 29.3%), Quartile 2 (n = 110; 28.42%), Quartile 3 (n = 101; 26.9%), and Quartile 4 (n = 116; 30.5%). By contrast, cardiovascular mortality gradually increased across the four quartiles. In subjects with the highest total IgE (n = 30; 7.9% in Quartile 4), their proportion of cardiovascular mortality was twice that of the subjects with the lowest total IgE (n = 14; 3.9% in Quartile 1). With regard to mortality risk, no difference was observed in all-cause mortality with an increase in total IgE quartiles. However, subjects with the highest total IgE (Quartile 4) had adjusted HR of 3.19 (95% CI: 1.71–5.96) cardiovascular mortality compared with the reference group (Quartile 1). In the Kaplan–Meier survival plots (Figure 2), the first graph refers to the survival curve for all-cause mortality and the graph below for cardiovascular mortality. Elevated total IgE levels were significantly associated with increased cardiovascular mortality (Log-rank test: *p*-value = 0.0005), but not all-cause mortality.

Table 3 shows HRs (95% CI) for all-cause and cardiovascular mortality with regard to total IgE quartiles, according to the history of baseline cardiovascular diseases. With regard to total IgE quartiles, overall non-linear association was observed with the risk of all-cause and cardiovascular disease mortality. For subjects with cardiovascular diseases at baseline, increases in total IgE levels were not associated with the risk of all-cause and cardiovascular mortality (all *p*-values > 0.05). In contrast, subjects without cardiovascular diseases at baseline showed greater risk for cardiovascular mortality in Quartile 2 (adjusted HR = 3.43; 95% CI: 1.08–10.94) and Quartile 4 (adjusted HR = 6.51; 95% CI: 1.81–23.43).

## 4. Discussion

Using a prospective US representative cohort, a significant association was observed between total serum IgE levels and cardiovascular mortality after a median follow-up of 119 months. Participants with elevated total IgE levels (≥123.01 kU/L in Quartile 4) had a 3.19-fold higher cardiovascular mortality risk than those with low total IgE levels (≤16.80 kU/L in Quartile 1). Subjects without history of baseline cardiovascular diseases (i.e., heart failure, coronary heart disease, heart attack, and angina) were at greater risk of cardiovascular mortality in terms of increases in the total IgE levels. Taken together, total IgE levels did not significantly predict overall mortality, but the elevation of total serum IgE levels may be a risk factor for cardiovascular mortality among older adults (≥50 years).

To the best of our knowledge, no previous studies have investigated the association between cardiovascular mortality risk and total IgE levels. The findings of this study are consistent with results of previous studies highlighting the IgE-mediated association between cardiovascular diseases and subsequent events [4]. Criqui et al. (1987) reported the first results in a population-based study showing a significant positive association between total IgE levels and cardiovascular diseases (i.e., previous myocardial infarction, stroke, and noninvasively diagnosed large-vessel peripheral arterial disease) in males [15]. Subsequent cross-sectional studies reported increased total IgE levels in patients with AMI, coronary heart disease, and angina compared with healthy counterparts or controls [8,9,10,16]. Using the same data from NHANES 2005–2006 as in the present study, Shiue et al. (2013) investigated the effect of total serum and 19 allergen-specific IgE levels with regard to self-reported cardiovascular events, including coronary heart disease, stroke, heart failure, heart attack, and angina [14]. The study concluded that total IgE and shrimp IgE were significantly associated with increased odds for coronary heart diseases (odds ratio (OR) = 2.02; 95% CI: 1.001–4.08 for total IgE and OR = 2.96; 95% CI: 1.06–8.28 for shrimp IgE), although its causality remained unclear [14]. Recently, Guo et al. (2016) measured total serum IgE levels among 708 patients admitted to a coronary angiography laboratory and observed a significant association between total IgE and severity of coronary artery disease (assessed by the number of diseased vessels showing ≥50% diameter stenosis and quantified using the Gensini score) [17]. After adjustment for traditional cardiovascular risk factors, elevated total IgE levels were strongly associated with an increased multi-vessel disease risk (OR = 1.003; 95% CI: 1.001–1.004). A significant linear association between total IgE quartiles and the Gensini score was observed (*p* for linear trend <0.0001) [17].

A significant association between elevated IgE levels and some forms of cardiovascular diseases appear to be prospectively confirmed. Langer et al. (1996) conducted a prospective study with 621 subjects (278 males and 343 females) for approximately 9 years. They found a significantly increased ischemic heart disease risk (relative risk (RR) = 3.4; 95% CI: 1.4–8.4) and non-fatal AMI development (RR = 7.3; 95% CI: 2.2–23.9) in males with baseline IgE levels of ≥200 kU/L compared with those with low baseline IgE levels) [12]. A study by Erdogan et al. (2003) further supported the clinical role of IgE in acute coronary syndrome [11]. IgE levels in 55 patients with AMI, unstable angina pectoris, and stable angina pectoris were consecutively measured on days 1, 3, 7, and 21 following admission and 3 months later and compared with those in 15 healthy controls. IgE levels were significantly increased during the acute phase of coronary syndromes and then gradually decreased, suggesting that an acute inflammatory response and mast cells are involved in plaque rupture [11]. Sinkiewicz et al. (2007) investigated whether IgE was involved in the atherothrombotic process [18]. In 80 patients with AMI, constant positive correlations among IgE concentrations of >100 kU/L (measured 7, 14, and 40 days after infarction), thrombogenesis markers, lipid parameters, and lipoprotein levels were detected.

Despite scientific evidence showing the function of total IgE in increased cardiovascular events, the causality dilemma is as baffling as the chicken and egg problem [4]. In particular, whether elevated IgE levels precede cardiovascular events remains unclear. Current study findings (Table 3) may offer useful insights into this dilemma. In subjects without baseline cardiovascular diseases, the elevation of total IgE was significantly associated with increased cardiovascular mortality risk, whereas no association was found in subjects with baseline cardiovascular diseases. Considering that IgE activates an increasing number of mast cells, interact with immune cells (i.e., monocytes and macrophages), and influence generic determinants of atherosclerotic disease [5,6,7,19], IgE levels in subjects who are free of clinical cardiovascular disease may be an important marker for subsequent cardiovascular events. In patients who already have cardiovascular diseases, on the other hand, IgE increase may be not specific for predicting cardiovascular mortality risk. Ultimately, our results support a potential causal inference, wherein elevated IgE levels contribute to proatherogenic and prothrombotic/antifibrinolytic actions [4]. Taken together, IgE levels may represent a risk factor for cardiovascular mortality.

This is the first study to report a significant association between total IgE levels and cardiovascular mortality risk based on the high-power and reliable nationwide NHANES (2005–2006) data. However, limitations of this study should be addressed. The NHANES Linked Mortality File provides mortality data from death certificates. This approach may overestimate the burden of cardiovascular events as the cause of mortality, particularly at older ages [20]. Therefore, a misclassification bias cannot be excluded. Furthermore, participants’ characteristics were only available only from the initial survey, and changes in health behaviors or cardiovascular risk factors from baseline were not considered. For example, total IgE was measured at the beginning of the 119 months’ follow-up study; thus, it is impossible to reflect any changes in health status over a 10-year testing period. Because of the study’s observational nature, the possibility of residual confounding effects due to unmeasured confounders cannot be excluded. Moreover, bias associated with several variables that were based on self-reported data must be considered.

## 5. Conclusions

In summary, we found that older adults with elevated IgE levels had a higher cardiovascular mortality risk than those with lower IgE levels. The risk was predominant in subjects without cardiovascular diseases at baseline than in those with baseline cardiovascular diseases. These findings suggest that elevated IgE levels may be a risk factor for increased cardiovascular mortality.

## Figures and Tables

**Table 1:** Characteristics of censored and deceased populations (n = 1496).

	No. of All Participants	Censored (n = 1065) No. (%)		Deceased (n = 431) No. (%)		*p*-Value
Age (year)						
50–59	425	390	(91.76)	35	(8.24)	<0.0001
60–69	472	383	(81.14)	89	(18.86)	
70–79	350	218	(62.29)	132	(37.71)	
≥80	249	74	(29.72)	175	(70.28)	
Sex						
Male	752	517	(68.75)	235	(31.25)	0.0362
Female	744	548	(73.66)	196	(26.34)	
Ethnicity						
White	847	555	(65.53)	292	(34.47)	<0.0001
Black	338	255	(75.44)	83	(24.56)	
Hispanic	279	227	(81.36)	52	(18.64)	
Others	32	28	(87.50)	4	(12.50)	
Family income ($)						
<20,000	484	292	(60.33)	192	(39.67)	<0.0001
≥20,000	1012	773	(76.38)	239	(23.62)	
Educational attainment						
High school or less	565	369	(65.31)	196	(34.69)	0.0002
High school graduate	482	351	(72.82)	131	(27.18)	
College or more	449	345	(76.84)	104	(23.16)	
Cigarette smoking						
Current smoker	303	217	(71.62)	86	(28.38)	0.0392
Former smoker	530	357	(67.36)	173	(32.64)	
Never smoked	663	491	(74.06)	172	(25.94)	
Moderate physical activity						
Yes	664	515	(77.56)	149	(22.44)	<0.0001
No	746	519	(69.57)	227	(30.43)	
Unable	86	31	(36.05)	55	(63.95)	
BMI (kg/m^2^)						
<18.5	18	9	(50.00)	9	(50.00)	0.0006
18.5–24.9	389	257	(66.07)	132	(33.93)	
25–29.9	517	361	(69.83)	156	(30.17)	
≥30.0	572	438	(76.57)	134	(23.34)	
History of disease						
Hypertension						
Yes	574	455	(79.27)	119	(20.73)	<0.0001
No	922	610	(66.16)	312	(33.84)	
Dyslipidemia						
Yes	802	581	(72.44)	221	(27.56)	0.2496
No	694	484	(69.74)	210	(30.26)	
Diabetes mellitus						
Yes	330	215	(65.15)	115	(34.85)	0.0061
No	1166	850	(72.90)	316	(27.10)	
Allergy history						
Yes	410	306	(74.63)	104	(25.37)	0.0707
No	1086	759	(69.89)	327	(30.11)	

*p*-values were calculated using the chi-square test.

**Table 2:** Number of events and adjusted hazard ratios for all-cause and cardiovascular mortality associated with total serum IgE levels at baseline.

	All-Cause Mortality			Cardiovascular Disease Mortality		
	No. Events (%)	Unadjusted HR (95% CI)	Adjusted HR (95% CI)	No. Events (%)	Unadjusted HR (95% CI)	Adjusted HR (95% CI)
Total IgE Quartile (kU/L)						
Quartile 1 (≤16.80)	105 (29.3)	1 [Reference]	1 [Reference]	14 (3.9)	1 [Reference]	1 [Reference]
Quartile 2 (16.81–44.10)	110 (28.42)	0.81 (0.61–1.07)	0.90 (0.71–1.14)	23 (5.9)	1.98 (0.90–4.36)	2.56 (1.10–5.94)
Quartile 3 (44.11–123.00)	101 (26.93)	1.01 (0.63–1.61)	1.04 (0.65–1.66)	18 (4.8)	1.24 (0.55–2.80)	1.51 (0.69–3.31)
Quartile 4 (≥123.01)	116 (30.45)	1.14 (0.83–1.56)	1.04 (0.64–1.69)	30 (7.9)	3.02 (1.77–5.13)	3.19 (1.71–5.96)

Adjusted HR was adjusted for age, sex, race/ethnicity, income, education, smoking status, moderate physical activity, BMI, history of hypertension, dyslipidemia, diabetes, and allergy.

**Table 3:** Number of events and adjusted hazard ratios for all-cause and cardiovascular mortality associated with total serum IgE levels at baseline, according to the presence or absence of baseline cardiovascular disease.

	All-Cause Mortality			Cardiovascular Disease Mortality		
	No. Events (%)	Unadjusted HR (95% CI)	Adjusted HR (95% CI)	No. Events (%)	Unadjusted HR (95% CI)	Adjusted HR (95% CI)
***Subjects with Baseline Cardiovascular Disease***						
Total IgE Quartile (kU/L)						
Quartile 1 (≤16.80)	33 (47.14)	1 (Reference)	1 (Reference)	7 (10.00)	1 (Reference)	1 (Reference)
Quartile 2 (16.81–44.10)	26 (41.27)	0.83 (0.37–1.82)	1.37 (0.60–3.15)	9 (14.29)	2.14 (0.70–6.52)	3.65 (0.87–15.33)
Quartile 3 (44.11–123.00)	34 (49.28)	1.01 (0.53–1.93)	1.65 (0.86–2.50)	10 (14.49)	0.99 (0.42–2.30)	2.16 (0.76–6.13)
Quartile 4 (≥123.01)	39 (53.42)	0.69 (0.38–1.28)	1.13 (0.62–2.05)	15 (20.55)	1.37 (0.69–2.69)	2.53 (0.88–7.24)
***Subjects without Baseline Cardiovascular Disease***						
Total IgE Quartile (kU/L)						
Quartile 1 (≤16.80)	72 (25.00)	1 (Reference)	1 (Reference)	7 (2.43)	1 (Reference)	1 (Reference)
Quartile 2 (16.81–44.10)	84 (25.93)	0.90 (0.61–1.35)	0.92 (0.66–1.30)	14 (4.32)	2.88 (0.80–10.36)	3.43 (1.08–10.94)
Quartile 3 (44.11–123.00)	67 (21.90)	1.00 (0.50–1.99)	0.98 (0.52–1.84)	8 (2.61)	1.65 (0.36–7.64)	1.95 (0.38–9.87)
Quartile 4 (≥123.01)	77 (25.00)	1.33 (0.91–1.95)	1.06 (0.55–2.04)	15 (4.87)	5.57 (1.86–24.61)	6.51 (1.81–23.43)

Adjusted HR was adjusted for age, sex, race/ethnicity, income, education, smoking status, moderate physical activity, BMI, history of hypertension, dyslipidemia, diabetes, and allergy.
